# Combining Oncolytic Viruses With Cancer Immunotherapy: Establishing a New Generation of Cancer Treatment

**DOI:** 10.3389/fimmu.2020.00683

**Published:** 2020-04-28

**Authors:** Tao Shi, Xueru Song, Yue Wang, Fangcen Liu, Jia Wei

**Affiliations:** The Comprehensive Cancer Centre of Drum Tower Hospital, Medical School of Nanjing University & Clinical Cancer Institute of Nanjing University, Nanjing, China

**Keywords:** oncolytic virus, cancer immunotherapy, checkpoint blockade, CAR-T, cancer vaccine

## Abstract

The recent successes of tumor immunotherapy approaches, such as immune checkpoint blockade (ICB) and chimeric antigen receptor T cell (CAR-T) therapy, have revolutionized cancer treatment, improving efficacy and extending treatment to a larger proportion of cancer patients. However, due to high heterogeneity of cancer, poor tumor cell targeting, and the immunosuppressive status of the tumor microenvironment (TME), combinatorial agents are required to obtain more effective and consistent therapeutic responses in a wide range of cancers. Oncolytic viruses (OVs) are able to selectively replicate in and destroy tumor cells and subsequently induce systematic anti-tumor immune responses. Thus, they are ideal for combining with cancer immunotherapy. In this review, we discuss the current understanding of OVs, as well as the latest preclinical and clinical progress of combining OVs with cancer immunotherapies, including ICB, CAR-T therapy, bispecific T cell engagers (BiTEs), and cancer vaccines. Moreover, we consider future directions for applying OVs to personalized cancer immunotherapies, which could potentially launch a new generation of cancer treatments.

## Introduction

Oncolytic viruses (OVs) have been selected or genetically engineered to preferentially infect, replicate in, and lyse tumor cells while sparing normal cells (1). The ability of OVs to enter and destroy cancer cells was first proposed more than a century ago, but the therapeutic benefits of OVs in cancer patients have only been recently realized in clinical trials, with the help of improving biological and genetical technology to generate more potent and tumor-specific viruses (2, 3). There has been increased interest in OVs due to a better understanding of viral biology mechanisms, tumor immunology, and molecular genetics. As a result, there has been vast development in OV therapies over the past two decades that has led to their increased specificity, potency, and tolerability, as well as multiple combination therapies that combine OV targeting with cancer immunotherapy (4–6).

Endowed with specific oncolytic properties, OVs may be natural or genetically engineered. Certain viruses, like myxoma virus and reovirus, are normally non-pathogenic in humans but have the ability to enter and selectively replicate within cancer cells (7). Many oncolytic viruses have been employed as genetic vectors to augment anti-tumor immune responses, including adenoviruses, herpes simplex virus type-1 (HSV-1), vesicular stomatitis virus (VSV), poliovirus, measles virus, Newcastle disease virus (NDV), reovirus, and others (8–10). As a result, many preclinical and clinical trials have demonstrated the anticancer potential of OVs among multiple cancer types, such as melanoma, glioblastoma, head and neck carcinoma, and colorectal cancer (11–13). Notably, in a landmark achievement for OV therapies, H101 (a genetically modified oncolytic adenovirus) received approval in 2005 in China to be combined with chemotherapy for the treatment of nasopharyngeal carcinoma, making H101 the world’s first OV to be used on cancer patients (14). Talimogene laherparepvec (T-VEC), a genetically modified HSV-1 encoding granulocyte-macrophage colony-stimulating factor (GM-CSF), was approved by the US Food and Drug Administration (FDA) in 2015 to treat patients with unresectable stage III and IV melanoma (15). Additionally, phase II or III trials for cancer immunotherapies combined with T-VEC or other OVs, such as Adv-TK, JX-594, and HSV1716, have shown clinical promise (16–18). Meanwhile, other ongoing clinical trials using OVs alone or combined with immune adjuvants to treat multiple cancer types such as glioblastoma (NCT03294486), colorectal cancer (NCT03206073), NSCLC (NCT03004183), and pancreatic cancer (NCT02705196) are under active or recruiting stage and are given high expectations to bring positive outcomes.

There are high expectations around harnessing the immune system to cure cancer, and the 2018 Nobel Prize in Physiology or Medicine was awarded to James Allison and Tasuku Honjo for their discovery of cancer therapy by inhibiting negative immune regulation. Currently, the major cancer immunotherapies are designed to activate innate or adaptive immune cells, like T and NK cells, in the tumor microenvironment (TME) to control tumor progress. They have shown promising results in preclinical and clinical trials (19–21). However, the long-term treatment effects of multiple immunotherapy approaches were not satisfactory in solid tumors. This may partly be due to the immunosuppressive status in the TME mediated by negative immune cells [such as tumor-associated macrophages (TAMs), T-regulation cells (Tregs), and myeloid-derived suppressor cells (MDSCs)] and immune inhibitory cytokines (such as IL-10, TGF-β, IL-35, and IL-27), and the poor tumor targeting and penetration of tumor-reactive immune cells (22, 23).

Considering the ability of OVs to selectively replicate in tumor cells and induce systematic immune responses, combining OVs with cancer immunotherapies could overcome immune inhibitions in the TME, and greatly improve anti-cancer therapeutic effects. In this review, we provide a thorough and comprehensive understanding of recent approaches combining OVs with multiple cancer immunotherapies that function via different treatment mechanisms, including checkpoint blockade therapy, adoptive cell transfer (ACT), bispecific T cell engagers (BiTEs) and cancer vaccines. Additionally, we discuss future developments in applying OVs to personalized cancer immunotherapies in order to achieve more accurate and effective treatment effects, potentially launching a new generation of cancer treatments.

## Two Basic Mechanisms of Oncolytic Virus Immunotherapy

The success of OV immunotherapy requires both selective tumor cell entry and the induction of systematic immune responses. Because of the inherent abnormalities in cancer cell stress responses, cell signaling, and homeostasis, OVs have the ability to selectively enter, replicate in, and destroy cancer cells while sparing normal cells (24, 25). A variety of signaling pathways usually involved in viral clearance, including IFN (interferon), TLR (Toll-like receptors), JAK–STAT (Janus kinase–signal transducer and activator of transcription), and PKR (double-stranded RNA-activated protein kinase) pathways, may be deficient or inhibited in cancer cells, allowing OVs to enter and survive in these cells (26–28). Additionally, cancer cells may overexpress several surface receptors, including CD46, ICAM-1 (CD54), DAF (CD55), CD155 and integrins, which enable OVs to enter tumor cells in the TME (29–32). For example, intrathecal delivery of an oncolytic recombinant poliovirus (PVS-RIPO) was observed to increase the median survival time of transgenic mice with glioblastoma multiforme that expressed the human poliovirus receptor CD155 (33).

After entering into tumor cells, OVs could induce systemic (including both innate and adaptive) immune responses which function to eradicate cancer cells within the TME. The lysis of tumor cells could release PAMPs (pathogen-associated molecular patterns), such as viral nucleic acids and proteins, as well as DAMPs (damage associated molecular patterns), such as HSP (heat shock proteins) and HMGB1 (high mobility group box 1), which stimulate the innate immune response. NK cells and macrophages within the TME may recognize PAMPs/DAMPs through cell surface PRRs (pattern recognition receptors), resulting in the secretion of inflammatory cytokines like IFN-α, IFN-γ, TNF-α, IL-6, and IL-12, which could then induce anti-viral and anti-tumor immune responses and recruit other innate immune cells from peripheral lymphoid organs (34, 35). Additionally, the release of TAAs (tumor-associated antigens) or TSAs (tumor-specific antigens) after tumor cell lysis and the antigen presentation by APCs (antigen-presenting cells) lead to an adaptive immune response and activation of antigen-specific CD4^+^ and CD8^+^ T cells. These tumor-reactive T cells can then cause immunogenic cell death (ICD) in tumor cells, a mechanism that has been confirmed in several preclinical studies (36–38). However, the induction of innate and adaptive immune responses by OVs could be a double-edged sword. Excessive priming of systematic antiviral responses could block OV replication and ongoing infection of tumor cells (39, 40). Thus, the appropriate timing of OV administration should be taken into consideration and the therapeutic outcome of OVs depends on a complex balance between anti-viral and anti-tumor immune responses. Moreover, although OV therapy alone has shown positive outcomes in some clinical trials, the systematic viral clearance and poor tumor targeting and infectivity of OVs remain major challenges for OV therapies, suggesting that further combination improvements are still needed to increase patient long-time benefit. In some cases, OVs could quickly turn into an exhausted status and failed to maintain continuous replication and lytic activity, which also highlighted the significance of OV combination with other cancer immunotherapies for a lasting anti-tumor immune responses (36, 41, 42).

## Combining Oncolytic Viruses With Cancer Immunotherapies

Based on the two major mechanisms underlying oncolytic virotherapy, OVs have the potential to induce T cell priming and infiltration, activate local immune responses, and counteract cancer-mediated immune evasion in the TME. Thus, OVs represent an ideal therapeutic platform to combine with cancer immunotherapies and potentiate treatment effects in multiple cancer types (36).

### Combining OVs With Immune Checkpoint Blockade Therapy

Immune checkpoint blockade (ICB) therapy aims to interrupt immunosuppressive tumor signals and restore anti-tumor immune responses by targeting checkpoint receptors or ligands, such as PD-1 (programmed cell death protein 1) and its ligand PD-L1, CTLA-4 (cytotoxic T lymphocyte-associated protein 4), LAG-3 (Lymphocyte-activation gene 3), and TIGIT (T-cell immunoreceptor with Ig and ITIM domains) (43, 44). ICB antibodies, such as Ipilimumab (anti-CTLA-4) and nivolumab (anti-PD-1), have demonstrated significant increases in objective response rates (ORRs) and overall survival compared with standard-of-care therapies in various solid tumor types (45–47). However, patient response rates were still very low due to low levels of TILs (tumor-infiltrating lymphocytes), low tumor mutational burden, and the lack of expression or presentation of TAAs/TSAs in the TME (48). The combination of OVs with several ICB therapies has provided a promising approach to overcome these limitations.

**FIGURE 1:**
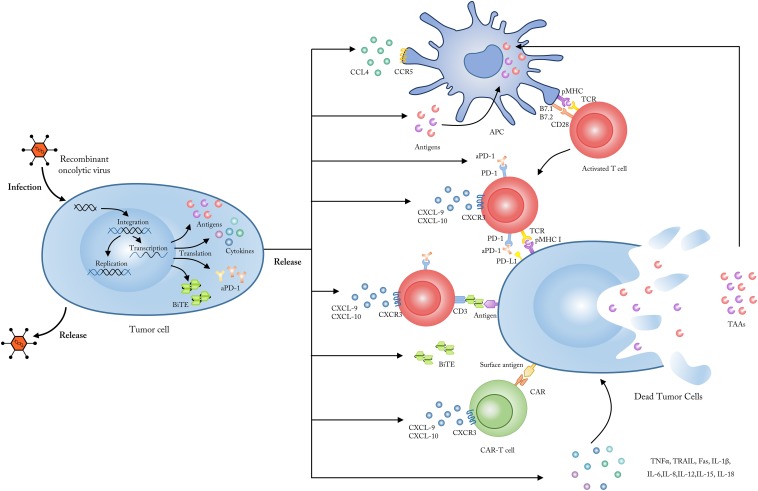
Employing OVs as genetic vectors to encode and secret targeted molecules. Upon entering into tumor cells, OVs could be modified to release, and secret several specific molecules including tumor antigens, which could be up-taken by APCs and subsequently presented to tumor-reactive T cells; chemokines like CXCL9 and CXCL10 which could enhance the penetration and activation of CAR-T cells; cytokines like TNF-a, IL-2, IFN-γ, IL-6, and IL-12 which could improve the anti-tumor immune responses and reverse the immunosuppressive status in TME; checkpoint antibodies which could inhibit the T cell immune tolerance mediated by immune checkpoints such as PD-1 and CTLA-4; BiTEs which bind to CD3 and a specific tumor antigen to improve the targeting and activation of antigen-specific T cells. CXCL, CXC-chemokine ligand; IL, interleukin; TNF, tumor necrosis factor; IFN, interferon; APC, antigen presenting cells; TCR, T cell receptor; MHC, major histocompatibility complex.

The combination of OVs with antibodies against multiple immune checkpoints have been investigated in multiple clinical trials (Table 1), among which the PD-1/PD-L1 and CTLA-4 combination therapies have advanced the furthest (49). As shown in Figure 1, the synchronous combinations of immune checkpoint antibodies with unmodified OVs or OVs armed with assisted cytokines and chemokines, such as TNF-a, IL-2, IL12, IL-15, GM-CSF, and IFN-β, have demonstrated synergistic therapeutic effects among metastatic or locally unresectable tumors (50–58). Firstly, for CTLA-4 combination, a randomized, open-label phase II study carried out by Chesney et al. in 2018 evaluated the efficacy and safety of a GM-CSF (granulocyte-macrophage colony-stimulating factor) encoding T-VEC combined with CTLA-4 targeting Ipilimumab among patients with locally unresected melanoma. Researchers found that Ipilimumab combined with T-VEC significantly improved the ORRs in both injected lesions and visceral lesions compared with Ipilimumab alone (39% vs. 18%, *P* = 0.002) (51). Promising ORR results (50%) were also observed among treatment of T-VEC plus Ipilimumab in a phase Ib trial for unresectable stage IIIB-IV melanoma patients (57). Secondly, for OVs combined with PD-1/PD-L1 blockade, Cervera et al. reported a preclinical study that concomitant delivery of adenoviruses armed with TNF-a and IL-2 and PD-1 blocking antibodies resulted in complete tumor regression in the B16. OVA melanoma mouse model (52). Also in 2017, Ribas et al. reported in a phase 1b clinical trial that the oncolytic virotherapy with T-VEC increased CD8^+^ T cell numbers and elevated PD-L1 protein expression, which improved the efficacy of pembrolizumab treatment and obtained an ORR of 62% (58). Furthermore, preclinical and clinical evidence has demonstrated that OVs may also be used as neoadjuvant agents to sensitize and improve therapeutic effects of subsequent tumor resection and ICI therapy. A preclinical study published in by Bourgeois et al. and a window-of-opportunity clinical study published by Samson et al. both in 2018 demonstrated that the early delivery of oncolytic Maraba rhabdovirus and reovirus coupled with subsequent surgical resection and PD-1 inhibitors provided increased cytotoxic T cell tumor infiltration and long-term survival benefits in a refractory TNBC (triple-negative breast cancer) mouse model and brain tumor patients (59, 60). This highlights the therapeutic potential of delivering OVs during pre-operative administration and combining OVs with post-operative ICIs. Considering the administration timing and sequence of OVs and other treatment approaches have a significant impact on therapeutic effects of such combinations, more research are needed to determine whether delivering OVs pre-operatively or combining OVs with post-operative ICIs or both for each specific patient.

**TABLE 1:** Current clinical trials of OVs combined with ICIs.

OVs	Genetic modifications	Checkpoint inhibitors	Phase	Cancer types	NCT number
HSV-1	T-VEC (Deletions in ICP34.5 and	Ipilimumab (anti-CTLA4)	I/II	Melanoma	NCT01740297
	ICP47 and transgenic expression of	Pembrolizumab (anti-PD1)	III	Stage IIIB–IV melanoma	NCT02263508
	GM-CSF)	Pembrolizumab	II	Stage III–IV melanoma	NCT02965716
		Pembrolizumab	I	HNSCC	NCT02626000
		Nivolumab (anti-PD1)	II	Lymphoma and non-melanoma skin cancers	NCT02978625
	Spontaneous deletion in the UL56	Ipilimumab	II	Melanoma	NCT02272855
	promoter	Ipilimumab	II	Melanoma	NCT03153085
Vaccinia virus	Deletions in thymidine kinase and	Ipilimumab	I	Advanced-stage solid tumors	NCT02977156
	transgenic expression of GM-CSF	Durvalumab (anti-PD1)/Tremelimumab (anti-CTLA4)	I	CRC	NCT03206073
	andβ-galactosidase (Pexa-Vec)	Nivolumab	I/II	HCC	NCT03071094
		Cemiplimab (anti-PD1)	I	RCC	NCT03294083
Coxsackie virus	None (CAVATAK)	Pembrolizumab	I	Melanoma	NCT02565992
		Pembrolizumab	I	NSCLC and bladder cancer	NCT02043665
		Ipilimumab	I	Melanoma	NCT02307149
		Ipilimumab	I	Melanoma	NCT03408587
Adenovirus	Engineered oncolytic adenovirus expressing GMCSF (ONCOS-102)	Pembrolizumab	I	Advanced or unresectable melanoma	NCT03003676
	p53 transduced adenovirus	Pembrolizumab	I/II	Head and neck cancer	NCT02842125
	(Ad-p53)	Nivolumab	II	HNSCC	NCT03544723
	Adenovirus vaccine expressing	Pembrolizumab	I/II	NSCLC	NCT02879760
	MAGE-A3 (Ad-MAGEA3)	Pembrolizumab	I	Metastatic melanoma and Squamous cell skin carcinoma	NCT03773744
Reovirus	None (Reolysin)	Pembrolizumab	I	Advanced pancreatic adenocarcinoma	NCT02620423
		Nivolumab	I	Relapsed/refractory multiple myeloma	NCT03605719
Vesicular Stomatitis	VSV-hIFNbeta-sodium iodide	Avelumab	I	Malignant solid tumor	NCT02923466
virus (VSV)	symporter [NIS] (VSV-IFNβ-NIS)	Pembrolizumab	I	Refractory NSCLC and HCC	NCT03647163

*HSV-1, herpes simplex virus-1; HNSCC, head and neck squamous cell carcinoma; CRC, colorectal cancer; HCC, hepatocellular carcinoma; RCC, renal cell carcinoma; NSCLC, non-small cell lung cancer.*

Apart from the direct combination of OVs with checkpoint antibodies, preclinical evidence indicated that OVs can also be engineered as genetic platforms to encode and secrete checkpoint antibodies within the TME (61–63). Engeland et al. generated attenuated Measles virus (MV) vectors encoding antibodies against both CTLA-4 and PD-L1 and found that the combination group demonstrated higher rates of complete tumor remission (>80%) than control vectors encoding anti-CTLA-4 and anti-PD-L1 in murine models of malignant melanoma (61). Additionally, in 2017, Bartee et al. generated a novel recombinant myxoma virus (vPD1) that could secrete a soluble form of PD1 from infected B16/F10 melanoma cells. vPD1 induced and maintained anti-tumor CD8^+^ T cell responses and was proven to be safer and more effective than the combination of systemic αPD1 antibodies with unmodified myxoma virus (62). However, many tumor types would require alternative combinations of OVs that target other immune checkpoints beyond PD-1/PD-L1 or CTLA-4. Currently, OVs targeting additional immune checkpoints including ICOS (inducible T cell co-stimulator), GITR (glucocorticoid-induced TNF receptor-related protein), OX40, TIGIT, and VISTA (V-domain immunoglobulin suppressor of T cell activation) are under active clinical investigation to enhance both viral and checkpoint blockade therapies (64–67).

Although combining OVs with immune checkpoint inhibitors (ICIs) is an appealing approach, this combination may also have antagonistic effects that should be considered. As described above, one issue is that the excessive priming of systematic antiviral responses could block OV replication and ongoing infection of tumor cells. But a preclinical study confirmed that viral oncolysis strongly induced PD-L1 expression in primary liver tumors and lung metastasis, resulting in the complete inhibition of tumor cell dissemination and abrogating resistance to PD-1 blockade therapy (68). Thus, the counter-regulator immune response of viral infection could also synergize with ICIs. Another major challenge is to ensure such combination therapy could have systematic treatment effects. Currently it remains difficult to make OVs reach every primary and metastatic tumor site to achieve the desired effects; also ICIs are most effective in patients with pre-existing T cell infiltrations, which are found mostly within the primary tumor lesion (69). Thus, for distant metastatic lesions with low T cell infiltration, treatment effects of OVs combined with ICIs could be very limited. Therefore, it is necessary to continue to search for more accurate and efficient immune modulatory factors to control OV-mediated anti-tumor T cell responses. For example, OV engineered to target PGE2 expressed by MDSC in TME could overcome localized immunosuppression and abrogated tumor resistance to PD-1 blockade therapy (70).

### Combining OVs With CAR-T Therapy

Chimeric antigen receptor T cell therapy involves designing, modifying, and amplifying T cells *in vitro* to grant them the ability to recognize tumor cell surface antigens via the transduced CAR structure on the T cell surface. This allows the CAR-T cells to enter the TME and kill tumor cells with corresponding specific antigens (71). Together with ICB therapy, CAR-T cells have revolutionized treatments for patients with previously refractory hematological cancers such as acute lymphoblastic leukemia (ALL) and chronic lymphocytic leukemia (CLL). CD19-specific CAR-T cell products were approved by the FDA in 2017 for the treatment of refractory B-cell lymphomas (72–77). However, only minor and transient ORRs were observed in patients with multiple solid tumors, potentially resulting from poor penetration of CAR-T cells into the TME and impaired CAR-T cell effector function in “cold” tumors (78–80). Thus, new combinatorial approaches that can overcome these barriers are urgently needed to enhance therapeutic outcomes of CAR-T cell therapy in both hematological and solid tumors.

**FIGURE 2:**
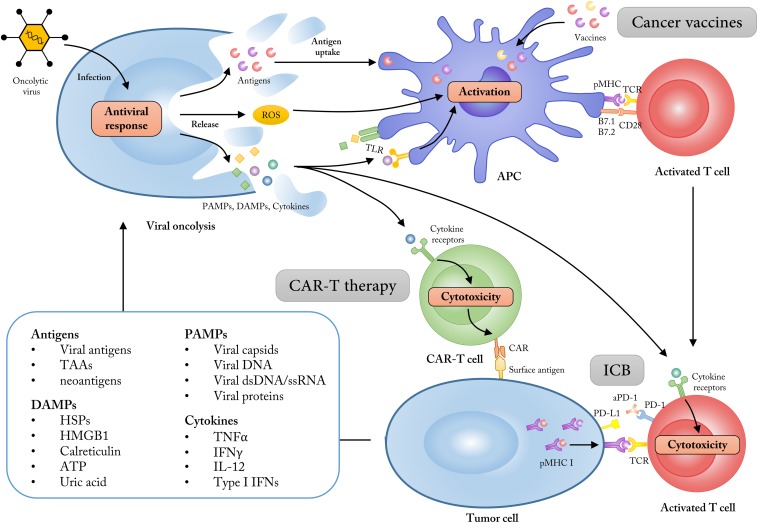
Employing OVs as adjuvants to synergize with multiple cancer immunotherapies. The ability of tumor cell selectivity and induction of systematic immune responses allow OVs as immune adjuvants to enhance the treatment effects of cancer immunotherapies like cancer vaccines, CAR-T therapy and immune checkpoint blockade (ICB). The lysis of tumor cells mediated by OVs could increase the release of tumor antigens, PAMPs, DAMPs, and some immune-stimulatory cytokines, which subsequently turn the “cold” tumor into “hot” tumor for immunotherapy approaches. TAAs, tumor-associated antigens; DAMPs, damage associated molecular patterns; HMGB1, high mobility group box 1; HSP, heat shock protein; PAMPs, pathogen-associated molecular patterns; dsDNA, double-stranded DNA; ssRNA, single-stranded RNA; ROS, reactive oxygen species; TLR, Toll-like receptor.

As described above, the OV-induced viral infection and the subsequent ICD of tumor cells make OVs excellent potential partners to synergize with CAR-T therapy (Figure 2). Indeed, several types of OVs have been engineered to deliver immunostimulatory cytokines, T-cell attracting chemokines, or even molecules targeting immune checkpoints in preclinical studies, which could promote migration, proliferation, and activation of CAR-T cells in solid tumors (81–87). Recently, an oncolytic adenovirus expressing TNF-α and IL-2 (Ad-mTNFα-mIL2) was combined with mesothelin-redirected CAR-T cell (meso-CAR-T) therapy to treat human-PDA (pancreatic ductal adenocarcinoma)-xenograft immunodeficient mice. Researchers found that Ad-mTNFa-mIL2 increased both CAR-T cell and host T cell infiltration into immunosuppressive PDA tumors and altered immune status in the TME, causing M1 polarization of macrophages and increased dendritic cell (DC) maturation (87). Additionally, Moon et al. intravenously administered a modified oncolytic vaccinia virus (VV.CXCL11) engineered to produce CXCL11 (a ligand of CXCR3) with the aim of increasing T cell trafficking into tumors in a subcutaneous tumor-bearing mouse model. VV.CXCL11 demonstrated the ability to recruit total and antigen-specific T cells into the TME after CAR-T cell injection and significantly enhanced anti-tumor efficacy compared with direct delivery of CXCL11 by CAR-T cells (82). In a slightly different approach, OVs can be engineered to produce antibodies against immune checkpoints in order to enhance CAR-T cell effects. In 2017 Tanoue et al. generated an oncolytic adenovirus with a helper-dependent Ad (HDAd) that expressed a PD-L1 blocking mini-antibody (CAd-VECPDL1). The combination of HER2-specific CAR-T cells with CAd-VECPDL1 showed improved anti-tumor activity and controlled tumor growth significantly better than either PD-L1 antibody or CAR-T cells alone in an HER2(+) prostate cancer xenograft model (86). Further, co-expression of a PD-L1 blocking antibody and IL-12p70 by oncolytic adenoviruses caused rapid tumor regression in head and neck squamous cell carcinoma (HNSCC) xenograft models when combined with HER2-specific CAR-T cells (84). Taken together, future clinical combination tests of OVs and CAR-T therapy could be highly expected to bring promising results. Additionally, several immune co-stimulatory molecules like OX40L, 4-1BBL (CD137), GITRL, and CD40L could enhance local activation and expansion of effector immune cells within the TME (65, 88–90). Thus, the combination of CAR-T cells with OVs that deliver immune co-stimulatory molecules is also promising.

As mentioned above, although engineered OVs are effective for delivering targeted molecules, systematic viral clearance and poor tumor targeting and infectivity of OVs remain major challenges for OV involved therapies. A different approach that attempts to overcome this involves using CAR-T and other antigen-specific T cells to deliver OVs to the TME (91–94). Loading CAR-T cells with low doses of viruses did not impact CAR receptor expression or function, and they could release and deposit viruses onto a variety of tumor targets before being neutralized by antiviral molecules (94). However, this combination approach is still very preliminary and more research is needed to evaluate the safety and efficacy of OV-delivering CAR-T products. Taken together, OVs and CAR-T cells are quite complementary; OVs can enhance tumor penetration and activity of CAR-T cells, while CAR-T cells can improve the subsequent oncolysis mediated by OVs. Moreover, combining OVs with other adoptive T cell products like TCR-T or NRT (neoantigen-reactive T cells) would also be a promising research direction.

### Combining OVs With BiTEs

Bispecific T cell engagers use DNA recombination technology to form bispecific antibodies by linking two specific single-chain antibody single-chain variable fragment (scFvs) via a ligation peptide. One of the BiTE scFvs can specifically bind to TAAs on the tumor cell surface, while the other scFv binds to CD3 or other T cell activators on the T cell surface, thereby improving the targeting, proliferation, and activation of tumor-reactive T cells in the TME (95, 96). BiTEs have shown impressive results in treating hematological malignancies, and in 2015, Blinatumomab, a bispecific T cell engager antibody targeting CD19 and CD3, was approved by the FDA for the treatment of Ph-R/R B-ALL (Philadelphia chromosome-negative precursor B-cell acute lymphoblastic leukemia) (97–100). However, similar to CAR-T therapy, application of BiTEs in solid tumors has been limited by low tumor penetration and off-target effects. The combination of BiTEs with OVs is being considered to enhance therapeutic efficacy, including employing OVs as genetic vectors to secrete BiTEs localized within the TME and break the tumor cell-mediated immunosuppressive status (101).

As shown in Figure 1, currently several targets of OV-delivered BiTEs have been tested in multiple types of hematological and solid tumors reported by several preclinical researches, including EGFR/CD3, EpCAM/CD3 (epithelial cell adhesion molecule), and EPHA2/CD3 (ephrin A2) (102–107). In 2017, an oncolytic adenovirus ICOVIR-15K was engineered by Fajardo et al. to express EGFR/CD3-targeting antibodies (ICOVIR-15K-cBiTE) in human lung and colorectal carcinoma xenograft mouse models. The results suggested that tumor-infiltrating T cells could be more effectively activated and redirected by ICOVIR-15K-cBiTE, compared with treatment by ICOVIR-15K alone (103). Also in 2017, another preclinical study by Freedman et al. reported that an adenovirus expressing EpCAM/CD3–specific BiTEs could improve the penetration and activation of both CD4^+^ and CD8^+^ T cells, thus enhancing T cell-mediated tumor killing in clinical tissue biopsy samples containing EpCAM-positive tumor cells (105). Considering that both BiTEs and CAR-T therapies are modified to target specific tumor antigens, a combinatorial approach using CAR-T cells and OVs armed with BiTEs has also been explored. In 2018, Wing et al. generated an oncolytic adenovirus armed with an EGFR-targeting BiTE (OAd-BiTE) combined with EGFR-targeting CAR-T cells in their preclinical study (108). They demonstrated that OAd-BiTE-mediated oncolysis greatly improved EGFR-targeting CAR-T cell activation and proliferation, which improved antitumor efficacy and prolonged survival in mouse models of colon and pancreatic cancer compared with monotherapy. Therefore, either double combination of OVs and BiTEs or triple combination of OVs, BiTEs, and CAR-T could have promising therapeutic potential in various solid tumors. Further clinical trials are needed for such combination therapy to be applied to a broader range of cancer patients.

Recently, there have been efforts to expand the therapeutic applications of BiTEs, leading to the conceptualization of bispecific/trispecific killer cell engagers (BiKEs/TriKEs) (109–112). Gleason et al. successfully generated a CD16-CD33 BiKE that aimed to induce NK (natural killer) cell function in 67 MDS (Myelodysplastic syndromes) patients. The CD16-CD33 BiKE significantly enhanced TNF-α and IFN-γ production and reversed CD33^+^ MDSC immunosuppression of NK cells in these MDS patients (109). Most recently, a novel type of trispecific NK cell engager was designed by Gauthier et al. to target two activating receptors (NKp46 and CD16) on NK cells and a tumor antigen on cancer cells. This proved to be more potent *in vitro* than clinical therapeutic antibodies targeting the same tumor antigens and could efficiently control tumor growth in mouse models of both solid and invasive tumors (110). In order to fully exploit OVs as genetic carriers, it may be feasible and more advantageous to use OVs to deliver BiKEs or TriKEs *in vivo* in order to further activate tumor-killing immune cells.

### Combining OVs With Cancer Vaccines

Cancer vaccines are designed to induce or amplify pre-existing cellular and humoral immune responses against target TAAs or TSAs (tumor neoantigens) *in vivo*, subsequently forming long-term immune memory to control tumor growth and prevent recurrence (20, 113). Presently, various types of tumor antigen-based platforms, including DNA, RNA, DC, and peptide cancer vaccines, have been investigated in clinical trials for the treatment of patients with multiple solid tumors, such as melanoma, colon carcinoma, sarcoma, and glioma (114–119). However, a deficiency of MHC II epitope presentation on lymph node-resident DC surfaces and the failure to recruit sufficient Th (T helper) cells to support the amplification of tumor-reactive CTLs remain major obstacles of vaccine-based cancer therapies (120, 121). Thus, the combination of OVs with tumor antigen-targeting vaccines has the potential to markedly activate and amplify tumor-reactive CTLs through OV-mediated immunoadjuvant effects or the mixture of MHC I cross-presented tumor epitopes and MHC II cross-presented OV epitopes (122).

Similar to the application of OVs as immunoadjuvants discussed above, OVs can also act as prime-boost regimens and synergize with tumor vaccines (Figure 2). The efficacy of this approach has been reported in several earlier preclinical studies (123–126). For example, the mixture of a DC1 (type-1-polarized DCs) vaccination and oncolytic vaccinia viruses expressing CCL5 (vvCCL5, whose receptors are expressed on CTLs induced by DC1) induced chemotaxis of lymphocyte populations both *in vitro* and *in vivo*, and showed enhanced therapeutic benefits in tumor-bearing mice vaccinated with DC1 and treated with vvCCL5 (123). Additionally, Woller et al. developed a tumor-targeted DC vaccine assisted by adenovirus and demonstrated that the intratumoral virus-induced inflammation could precondition the tumor for effective anti-tumor DC vaccination and elicit potent antitumoral CD8 + T cell responses in mice with lung cancer (124). Similar preclinical therapeutic results were also observed in combining a melanoma-targeting vaccine with GM-CSF armed VSV (126).

Another more developed combination strategy encoded OVs with one or more TAAs (shown in Figure 1), creating a so-called “oncolytic vaccine” that has been explored in both preclinical and clinical studies (127–132). For preclinical research, in 2016, Ragonnaud et al. generated a replication deficient adenovirus vaccine expressing the invariant chain (Ii) adjuvant fused to a TAA, which was then incorporated with the 4-1BBL membrane form to further activate CTLs. This oncolytic vaccine increased and prolonged TAA specific CD8 + T cells in tumor-bearing mice, and the local expression of 4-1BBL circumvented the toxicity associated with systemic antibody administration (128). Additionally, in 2018, a novel platform was developed by Ylosmaki et al. to attach tumor-specific peptides onto the viral envelope of vaccinia virus and HSV-1 (herpes simplex virus 1). The OVA SIINFEKL-peptide-coated viruses and gp100-Trp2-peptide-coated viruses induced strong T cell-specific immune responses toward these tumor antigens and enhanced treatment efficacy in B16. OVA and B16-F10 melanoma mouse models (131). As for early clinical evidence example, Galanis et al. reported a phase I trial that engineered oncolytic measles virus that expressed carcinoembryonic antigen (CEA) was well tolerated and resulted in improved median survival time for recurrent ovarian cancer patients (132).

Furthering the combination of OVs with cancer vaccines, a prime-boost oncolytic vaccine strategy has been evaluated in several preclinical studies that employed one viral vector for initial immune priming to then boost another viral vector encoding the same TAA (133–137). Adenovirus is the most common OV used for immune priming. In prostate cancer mouse models, a prime-boost viral-vector vaccination, which included an adenovirus ChAdOx1 and modified vaccinia Ankara virus MVA encoded with prostate cancer-associated antigens, induced strong antigen-specific CD8 + T cell responses and significantly improved survival in tumor-bearing mice when combined with a PD-1 blocking antibody (136). Additionally, the vaccination of adenovirus-boosted Maraba MG1 rhabdovirus, which both expressed the human MAGE-A3, led to an expansion of hMAGE-A3-specific CD4^+^ and CD8^+^ T cells persisting for several months in mouse models with MAGE-A3-positive solid malignancies (137). Investigations of clinical applications of this prime-boost oncolytic vaccine strategy are also under way (NCT02285816, NCT02879760, NCT03773744).

Currently, the targets of most OV-combined cancer vaccines are TAAs, which are overexpressed in tumor tissues but could also be expressed by normal tissues. Personalized cancer vaccines targeting tumor neoantigens (TSAs), which are detected only in tumor cells, are highly promising. Two recently co-published studies proposed a potential role for neoantigen-based vaccines in human glioblastoma treatment and demonstrated their ability to turn “cold” tumors, like glioblastoma, into “hot” tumors for subsequent tumor-killing CD8^+^ T cells (138, 139). Therefore, it is feasible to use viral vectors encoded with tumor neoantigens to further improve neoantigen presentation and T cell activation.

## Future Directions for OV-Combined Personalized Cancer Immunotherapy

In this review, we discussed four potential combination approaches of OVs with cancer immunotherapies, including checkpoint blockade therapy, CAR-T therapy, BiTEs, and cancer vaccines. To further the application of OVs in personalized cancer immunotherapy, we believe several future developments are needed.

First, the appropriate types of OVs should be selected depending on the specific combination strategy for each patient. For example, genetically simple and pro-inflammatory viruses, such as rhabdoviruses, are suitable for cancer vaccine combinations due to the rapid dissemination of such viruses in secondary lymphoid organ and the TME. Conversely, more complex and slower-replicating viruses, like adenovirus or vaccinia virus which could provide transgene expression persistently, are better suited to deliver checkpoint-blocking antibodies (53, 86, 140). Second, according to the tumor location and individual patient progress, conventional cancer treatments, including chemotherapy and radiotherapy, could be included in the OV-combined tumor immunotherapy to enhance synergistic effects of anti-tumor immune activation (141–143). Finally, considering that some biomarkers, such as MSI (Microsatellite instability), TMB (Tumor mutational burden), and PD-L1, have already been successfully used to predict and monitor PD-1 blockade therapy, it would be reasonable to evaluate these biomarkers or pursue other novel OV-specific biomarkers to better control and improve the OV-combined immunotherapy (144–147).

Apart from combining OVs with cancer immunotherapies, actually OVs have also been combined with standard cancer therapeutics like chemotherapy and radiotherapy. For example, in 2017 a phase I trial of intravenous oncolytic vaccinia virus combined with cisplatin and radiotherapy was shown to improve 1-year progress-free survival (74.4%) of locally advanced head and neck cancer patients (148). To make it even further, most recently Mahalingam et al. reported phase Ib single-arm study demonstrating that combination of oncolytic reovirus, pembrolizumab and chemotherapy showed encouraging treatment efficacy for patients with pancreatic ductal adenocarcinoma (PDAC) who progressed after first-line treatment (149). More promising results could be expected in the future with multiple combination of OVs, cancer immunotherapies and standard cancer therapies. In conclusion, OVs can be powerfully combined with existing cancer therapies, have the ability to accelerate cancer immunotherapy progress, and may launch a new generation of cancer treatment.

## Author Contributions

JW: conception and design. TS: manuscript writing. XS: figure preparing. YW and FL: review and editing. All authors read and approved the final manuscript.

## Conflict of Interest

The authors declare that the research was conducted in the absence of any commercial or financial relationships that could be construed as a potential conflict of interest.
